# Associations between biological and behavioral factors in early life and food consumption in Brazilian adolescents: Results from the ERICA study

**DOI:** 10.1371/journal.pone.0264714

**Published:** 2022-03-02

**Authors:** Maria Laura Siqueira de Souza Andrade, Juliana de Souza Oliveira, Poliana Coelho Cabral, Felipe Vogt Cureau, Vanessa Sá Leal, Pedro Israel Cabral de Lira

**Affiliations:** 1 Department of Nutrition, Universidade Federal de Pernambuco, Recife, Pernambuco, Brazil; 2 Nutrition Center, Centro Acadêmico de Vitória, Universidade Federal de Pernambuco, Vitória de Santo Antão, Pernambuco, Brazil; 3 Universidade Federal do Rio Grande do Sul, Porto Alegre, Rio Grande do Sul, Brazil; Addis Ababa University, ETHIOPIA

## Abstract

The aim of the present study was to determine associations between biological and behavioral factors in early life and food consumption in Brazilian adolescents. The sample was composed of 36,956 adolescents (12–17 years of age) who participated in the “Study of Cardiovascular Risk in Adolescents”. Sociodemographic, biological, and behavioral variables were collected using questionnaires self-administered by the adolescents. Early-life factors were assessed using a questionnaire administered to the parents/guardians of the adolescents. Dependent variables related to food consumption (total energy intake and percentages of macronutrient intake [carbohydrates, lipids and proteins]) were measured using the 24-hour recall method and compared to dietary reference intakes. Data analysis was performed with the aid of STATA 14.0, using multiple linear regression analysis with respective β coefficients. The level of significance was set at 5% (p ≤ 0.05). Adolescents born with low weight had lower energy intake (-94.8 kcal, 95%CI: -177.2; -12.3, p = 0.024) and 1.25% higher carbohydrate intake (95%CI: 0.15; 2.34, p = 0.025) compared to those born with adequate weight. Those who received exclusive breast breastfeeding for three to six months ingested 1.32% more lipids than those who received exclusive breast breastfeeding for less than three months (95%CI: 0.37; 2.26, p = 0.006). In conclusion, low birth weight was associated with lower energy intake and a higher percentage of carbohydrate intake, whereas breastfeeding three to six months was associated with a higher percentage of lipid intake.

## Introduction

Adolescence is a critical time for the establishment of health risk behaviors, such as inadequate food consumption [1], which can make eating behavior more difficult to modify in adulthood [2]. Inadequate food intake may be influenced by a set of interrelated factors occurring early in life, such as low birth weight [3], preterm birth [4], and breastfeeding duration, and may therefore affect subsequent stages of life [5].

The few studies that have examined the association between factors in early life and total energy and macronutrient intake in children and adolescents offer conflicting results [3, 6, 7]. Doornweerd et al. [8] found that adolescent twins with a lower birth weight had greater total energy intake (kcal/day) and saturated fat intake (g/day) in adolescence compared to those with a higher birth weight. In contrast, a cohort study found that young adults (19 to 27 years old) in the Philippines who had been born preterm with very low birth weight had a lower daily energy intake of macronutrients (carbohydrates, proteins, and fats) and micronutrients (calcium and vitamin D) [4]. Other studies also suggest that food preferences (increased intake of foods high in fat and carbohydrates) may vary according to sex and age in subjects born with underweight and/or with intrauterine growth restriction [9–11].

As a possible explanation, the influence of early life factors may be due to dysregulation of the energy balance (increase in food consumption and decreased levels of physical activity) and a consequent change in appetite. This can increase the preference for foods with high carbohydrate and lipid contents due to the physiological effects of these foods on the increase in satiety-related hormones (leptin and ghrelin) beginning in early life [12].

Regarding the breastfeeding pattern and duration, a study carried out with Japanese children found that those who were breastfed for ≥ six months had a lower probability of low vegetable intake than those breastfed for < six months [13]. Other studies have shown an association between longer breastfeeding and healthier eating behaviors in childhood and adolescence [13–15], such as an increase in the consumption of fruits and vegetables and a decrease in the consumption of processed foods [14]. However, the dependent variable analyzed in this study was related to the diet quality. These results lend strength to the hypothesis that breastfeeding can increase sensory experiences regarding new flavors for infants [16] and favor children’s acceptance of new foods [16], contributing to a more diversified eating behavior in the future [17].

Therefore, analyzing biological and behavioral factors in early life that may exert an influence on food consumption in adolescence is important [6], as food intake may be a mediating factor in the association between low birth weight and an increased risk of developing chronic noncommunicable diseases, such as obesity and cardiovascular disease [8, 12, 18, 19]. This issue requires further investigation to enable a better understanding of possible interrelations and mechanisms that are not yet fully known [6].

Moreover, studies indicate that the possible association between low birth weight and an increased risk of developing chronic noncommunicable diseases may be explained by unfavorable changes in the energy balance, which may be related to inadequate health behaviors, such as unhealthy food intake, physical inactivity, and sedentary behavior [4, 9]. The lack of a consensus on the association between factors established early in life and food consumption in later stages hinders the delineation of the problem in the introduction of this article, given that there is insufficient evidence in the literature on these interrelations and underlying mechanisms of early life factors that may explain such phenomena in humans [3, 4, 6]. Thus, the introduction of this study only indicates a theoretical assumption based on findings in the literature [6, 8].

Thus, the following was the guiding question of the present study: What biological and behavioral factors in early life are associated with food consumption and physical inactivity in Brazilian adolescents? This is an exploratory study in the field of health-related behaviors (food consumption) and the investigation of biological and behavioral factors to which adolescents are exposed in the first months of life, as exclusive breastfeeding, low birth weight, and preterm birth are independent of each other and express different biological or psychosocial phenomena. Assuming that these factors are independent, it is possible that they may be associated with food intake in different ways.

Therefore, the aim of the present study was to determine whether there are associations between biological (birth weight and duration of pregnancy) and behavioral (exclusive breastfeeding) factors in early life and food consumption in Brazilian adolescents.

## Methods

This study was conducted with data from the *Estudo de Riscos Cardiovasculares em Adolescentes* (ERICA [Study of Cardiovascular Risk in Adolescents]), which was a multicenter, school-based, cross-sectional study with a national scope. The sample was composed of adolescents 12 to 17 years of age enrolled at public and private schools in Brazilian cities with more than 100 thousand residents. The inclusion and exclusion criteria for the ERICA Study were determined by Bloch et al. [20].

The complex sampling process was stratified and performed in three stages, as described in the study by Vasconcellos et al. [21]. The population was divided into 32 geographic strata: all 26 capital cities, the Federal District, and five strata representing other municipalities with at least 100 thousand residents in each region of Brazil. Schools were selected based on probability proportional to size (number of students per school) and inversely proportional to the distance between the municipality of the school and the state capital. A total of 1,247 schools were selected from 124 municipalities. Three classrooms were randomly selected at each school and all students in these classrooms were invited to participate in the ERICA Study [20, 21].

For the calculation of the sample size on the national level, the expected prevalence of metabolic syndrome among adolescents was 4.0%, the maximum error was 0.9%, an 95% confidence interval was considered, and a design effect of 2.97 was used due to the complex cluster sampling (school, class, year, and shift). This calculation was based on the average body mass determined by processing data from the 2007 survey of the Vigilance of Risk Factors to the Health of Adolescents implemented by the City of Rio de Janeiro, Brazil [20, 21]. A detailed description of the sampling process can be found in the study by Vasconcellos et al. [21]. Thus, the size required for simple random sample would be 5,408 students, to which 15% was added to compensate for possible dropouts, leading to 6,219 adolescents. As the study should produce estimates with a specific precision for each of 12 domains (six ages and two sexes), a total sample of 74,628 adolescents would be needed. This number was rounded up to 75,060 adolescents, as multiples of 60 were needed in each stratum [21].

Among the total of adolescents considered eligible to participated in the ERICA Study (102,327), only 71,971 students completed the questionnaire designed for the study and the 25-hour recall. Moreover, 35,015 parents/guardians did not answer questions related to the onset of life, which was the main focus of the study. Therefore, the final sample comprised 36,956 adolescents [22, 23]. The characteristics of the sample are displayed in Table 1. The sample loss was 48.5%, which may have compromised the identification of some associations. [24] The main reason for the non-response rate was attributed to possible recall bias of the participants regarding variables related to the period of pregnancy [22, 24]. In an attempt to attenuate this limitation, categories without information were created for all variables with missing data. Moreover, the categories of variables that were significant may have been due to the robust, complex sampling of the ERICA Study, which enabled considerable variation in the distribution of data during the analysis. The authors decided not to impute missing data, as values predicted via imputation can greatly skew the results, according to Little and Rubin [24].

**Table 1:** Characterization of environmental, sociodemographic, biological, and behavioral variables in early life of Brazilian adolescents stratified by sex, ERICA 2013–2014.

Variables	Female Sex		Male Sex	
	%	CI_95%_	%	CI_95%_
**Regional Distribution**				
Central West	8.0	7.7–8.1	8.0	7.7–8.1
North/Northeast	27.0	26.8–27.2	27.1	27.0–27.4
South/Southeast	65.0	64.9–65.3	64.9	64.7–65.1
**Geographic Stratum**				
Capital	42.0	41.1–42.6	41.9	40.7–43.1
Instate Municipality	58.0	57.2–60.4	58.1	57.4–60.5
**Type of School**				
Public	78.0	72.5–82.7	77.3	71.9–81.9
Private	22.0	17.2–27.4	22.7	18.0–28.0
**Age Group (years)**				
12–14	46.2	46.0–46.4	47.0	46.7–47.1
15–17	53.8	53.6-54-0	53.0	52.9–53.3
**Sexual Maturity**				
Prepubescent	0.3	0.2–0.4	0.5	0.4–0.8
Pubescent	61.4	59.7–63.0	63.4	61.3–65.5
Postpubescent	38.3	36.5–39.9	35.8	33.8–38.0
No information	0.0	0.00–0.02	0.0	0.03–0.16
**Skin Color**				
Non-White	58.5	56.2–60.7	57.6	55.5–59.7
White	40.0	37.7–42.2	39.6	37.3–41.7
No information	1.5	1.1–2.0	2.8	2.3–3.3
**Socioeconomic Class**				
High	6.6	5.5–7.7	8.9	7.5–10.5
Middle	53.0	50.9–55.0	53.0	50.5–55.3
Low	8.3	7.2–9.4	5.3	4.4–6.2
No information	32.1	30.3–34.0	32.8	30.8–34.8
**Nutritional Status**				
Ideal range	73.9	71.8–75.8	72.5	70.3–74.5
Overweight	17.8	16.2–19.4	17.4	15.8–19.0
Obesity	8.3	7.4–9.1	10.1	9.0–11.3
**Birthweight (g)**				
Low	7.0	5.8–8.4	6.5	4.6–8.8
Insufficient	17.2	15.8–18.7	12.3	11.0–13.8
Adequate	42.9	40.4–45.3	43.2	40.7–45.7
High	10.3	9.2–11.5	12.5	11.2–13.8
No information	22.6	19.0–26.4	25.5	21.9–29.4
**Duration of Pregnancy (months)**				
≤ 8	7.7	6.9–8.5	7.6	6.7–8.5
9 to 10	69.6	65.9–72.9	66.0	62.4–69.2
No information	22.7	19.1–26.6	26.4	22.7–30.4
**Exclusive Breastfeeding (months)**				
< 3	12.0	10.8–13.1	11.3	10.1–12.7
3 to 6	43.1	40.9–45.2	42.0	39.2–44.7
>6	5.7	5.0–6.4	6.5	5.6–7.3
No information	39.2	36.4–42.0	40.2	37.0–43.4
**Practice of Physical Activity**				
Sufficiently Active	29.6	28.0–31.1	60.8	58.9–62.7
Insufficiently Active	70.4	68.8–71.9	39.2	37.2–41.0

CI: confidence interval; Socioeconomic class: High = subcategories A1 and A2; Middle = subcategories B1, B2, and C1; Low = subcategories C2, D, and E^14^. BMI/age: body mass index for age, classified as underweight (z-score <-2), ideal range (z-score ≥-2 and ≤+1), overweight (z-score >+1 and ≤ +2), obesity (z-score >+2), and severe obesity (z-score >+3)^17^; birthweight: low (<2500g); insufficient (2500 to 2999g); adequate (3000g to 3999g), and high (≥ 4000g)^15^. Duration of pregnancy: <8 months and 9 to 10 months. Duration of exclusive breastfeeding: < 3 months, 3 to 6 months, and > 6 months^5^.

Data collection was carried out between February 2013 and November 2014 by previously trained researchers using questionnaires self-administered by the parents or adolescents and an electronic aid (personal digital assistant) [20, 21]. Early-life factors were collected from the parents/guardians through a questionnaire sent to the homes and returned by the adolescents on the day of collection [20, 21].

The dependent variable was food consumption, which was collected using the 24-hour recall technique and “multiple-pass method”. In a face-to-face interview, the adolescent was asked about food consumed the previous day, how it was prepared, and respective portions with the aid of the ERICA-REC24 software described in the study by Barufaldi et al. [25]. To calculate intra-personal variation in food consumption, another 24-hour recall was applied to approximately 20% of the sample, which was used for the correction of the distribution and calculation of the usual intake of foods and nutrients.

The intake of energy (kcal/day) and macronutrients (carbohydrates, lipids, and proteins) was determined using the Nutritional Composition Table of Foods Consumed in Brazil [26] and Reported Measures Table for Foods Consumed in Brazil [27]. The nutritional recommendations for male and female adolescents (nine to 18 years of age) followed the guidelines of the Dietary Reference Intakes (DRIs) for macronutrients: 45 to 65% carbohydrates, 20 to 35% lipids, and 10 to 35% proteins^.^[28].

For the calculation of the Estimated Energy Requirement (EER), a standard adolescent (most frequent in the sample) was considered to have a median age of 15 years (interquartile range: 13 to 16) and was physically inactive, with no differences between the sexes. With this, the recommendation of the Institute of Medicine [28] is 1731 kcal for girls and 2223 kcal for boys.

The following were the covariables: 1) sex (male or female); 2) age (12–14 years or 15–17 years); 3) mother’s schooling (≤ 4 years; 5 to 8 years, or > 8 years); 4) family income *per capita* (sum of incomes of family members) and categorized using on the criteria proposed by the *Associação Brasileira de Empresas de Pesquisa* (ABEP [Brazilian Association of Research Firms]) [29], which classifies families as having high income (Classes A1 and A2), middle income (Classes B1, B2, and C1), or low income (Classes C2, D, and E); 5) type of school (public or private); 6) geographic stratum (capital city or instate municipality); 7) regional distribution (central west, south/southeast, or north/northeast).

The following biological factors related to early life were considered: 1) birth weight in grams (g) reported on the form and categorized using the cutoff points proposed by Puffer & Serrano: [30] low weight (< 2500 g), insufficient weight (2500 to 2999 g), adequate weight (3000 to 3999 g), and high weight (≥ 4000 g) [30]. Duration of pregnancy was identified by the reported number of months until birth and categorized as < 8 months or 9 to 10 meses.

The behavioral factor in early life was the duration of exclusive breastfeeding, which was identified by the time in months the newborn exclusively received mother’s milk at the breast and was categorized based on the classification proposed by Labayen et al. [19] < three months; three to six months; or > six months.

The stage of sexual maturity was self-reported by the adolescents with the use of figures based on the criteria proposed by Tanner [31] and the adolescents were categorized as prepubescent, pubescent, or postpubescent.

Nutritional status was determined using the body mass index (BMI = body mass [kg] / height [m]^2^) calculated with the aid of the WHO Anthro Plus software (2007), recommended by the World Health Organization (WHO, 2007) and the Brazilian Ministry of Health [32] for adolescents up to 19 years of age. Body mass was determined using an electronic scale (*Líder*) with a capacity of 200 kg and precision of 50 g. Height was determined using a portable stadiometer (*Alturexata*) with a capacity of 213 centimeters and precision of 1 mm. The adolescents were classified using the z-score cutoff points described by the WHO (2007): [32] Z-score < -2 (underweight); Z-score ≥ -2 and ≤ +1 (ideal range); Z-score +> 1 and ≤+2 (overweight); >+2 (obesity). The adolescents were subsequently categorized as without excess weight, with overweight, or with obesity [32]. Based on this reference, the authors opted for an operational classification of the variable, with individuals classified as having a z-score <-2 (underweight) and z-score ≥-2 and ≤+1 being considered without excess weight (normal weight). Adolescents with a z-score > +1 and ≤+2 were categorized as overweight and those with a z-score > +2 were categorized as obese.

Physical inactivity was determined from the answers to the adapted Self-Administered Physical Activity Checklist. Time and frequency per week (days, hours, and minutes) for each physical activity listed was quantified, followed by the calculation of the total time generated during these activities [33]. Adolescents who spent more than 300 minutes carrying out these activities were classified as “active”, whereas others were classified as “insufficiently active” [33].

Statistical analyses were performed with the aid of the STATA program (version 14.0) using the *“Survey”* module due to the complex sampling design of the ERICA Study. Continuous variables were tested for normality of distribution using the Kolmogorov-Smirnov test. Variables with non-Gaussian distribution were expressed as median and interquartile range (IQR). Subsequently, logarithmic transformation was performed for the data on energy and macronutrient intake.

Multiple linear regression was used to determine the relative contribution of the different percentages of energy and macronutrient intake (dependent variables) according to early life factors (independent variables) controlled for confounding variables, such as socioeconomic factors (mother’s schooling and economic class), geographic factors (regional distribution and geographic stratum), and biological factors (sex, age group, sexual maturity, and excess weight) of the adolescents. The stepwise backward method was used for the selection of variables in the model, adopting a p-value > 0.20 as the exclusion criterion. The results were expressed as beta coefficients (β) in the unadjusted and adjusted analyses with 95% confidence intervals (CI). In the final model, variables with a p-value ≤ 0.05 were considered significantly associated.

The ERICA study received approval from the institutional review board of each of the 27 units of Brazil. All adolescents and their legal guardians provided written consent to participate in the study.

## Results

For the development of the present study, the sample was composed of the set of adolescents who had complete data from the questionnaires administered to the guardians and adolescents themselves regarding biological and behavioral factors in early life (birth weight, preterm birth, and exclusive breastfeeding) as well as socioeconomic and lifestyle aspects. A total of 36,956 adolescents of all those who participated in the ERICA Study (71,971 adolescents) had complete data from the questionnaires and 24-hour recall and composed the present sample.

The 36,956 adolescents who participated in the present study represented a population of 6,628,961. The characterization of the sample stratified by sex is displayed in Table 1. The distribution of proportions was similar in terms of regional distribution and geographic stratum. However, higher frequencies were found of adolescents born with adequate weight, those born after nine or ten months of pregnancy, and those having been breastfed exclusively for three to six months (Table 1).

Mean age of the adolescents was 14.7 years (SD = 1.6). Most were in the older age group (53.4%), residents of instate municipalities (58.1%), and enrolled in the public school system (77.7%). Moreover, the highest proportion of students was distributed in the south/southeastern region of the country and the lowest proportion was distributed in the north/northeastern region.

Median energy intake was 2,545 kcal for insufficiently active boys (IQR: 1542.5 to 2820.7) and 1,731 kcal for insufficiently active girls (IQR: 1509.1 to 2127.5). For both sexes, the percentage contribution of macronutrients to the total energy value was 54.5% carbohydrates, 15.3% proteins, and 29.8% lipids.

The results of the multiple linear regression analysis of early life biological and behavioral factors, energy intake, and percentage of macronutrient intake (carbohydrates, proteins, and lipids) are shown in Tables 2 and 3. A negative association was found between birth weight and energy consumption, even after controlling for adjustment variables. Adolescents born with low weight had lower energy intake (-94.8 kcal, 95% CI: -177.2 to -12.3, p = 0.024) compared to their peers (Table 2). The adjusted model explained 7.4% of the energy consumption in adolescence.

**Table 2:** Multiple linear regression between biological and behavioral factors in early life and energy intake in Brazilian adolescents, ERICA 2013–2014.

Energy Intake (Kcal)					
Variables	Crude Analysis β (CI95%)	p	Adjusted Analysis* β (CI95%)	p	R^2^ (%)
**Birthweight (g)**					7.4**
Adequate	1			0.024	
Low	-52.1 (-152.7–48.3)	0.309	-94.8 (-177.2;-12.3)		
Insufficient	93.6 (-29.9–214.3)	0.275	131.2 (-25.8;288.4)	0.101	
High	92.2 (-29.9–214.3)	0.139	79.1 (-58.8;217.2)	0.261	
No information	-2.2 (-84.6–80.1)	0.957	-6.0 (-91.8;79.8)	0.891	
**Duration of Pregnancy (months)**					7.16***
≤ 8	1				
9 to 10	4.6 (-150.9;160.1)	0.954	-17.6 (-168.7;133.5)	0.819	
No information	-31.4 (-228.7;165.9)	0.755	-59.3 (-215.0;96.2)	0.454	
**Exclusive Breastfeeding (months)**					
< 3	1				
3 to 6	-40.5 (-130.2;49.1)	0.375	-59.3 (-146.1;24.4)	0.180	7.25****
> 6	62.6 (-60.0;185.2)	0.317	23.0 (-93.8;139.9)	0.699	
No information	-77.9 (-172.5;16.7)	0.106	-102.2 (-186.7;-17.8)	0.018	

* Analysis adjusted by variables: Regional Distribution, Geographic Stratum, Sex, Age Group, Nutritional Status, Practice of Physical Activity, and Sexual Maturity.

** Birthweight: R^2^ = 0.16; versus Regional Distribution: R^2^ = 0.44; versus Geographic Stratum: R^2^ = 1.56; versus Sex: R^2^ = 5.08; versus Age Group: R^2^ = 5.90; versus Nutritional Status: R^2^ = 7.24; versus Practice of Physical Activity: R^2^ = 7.36; versus Sexual Maturity: R^2^ = 7.40.

*** Duration of Pregnancy: R^2^ = 0.02; versus Regional Distribution: R^2^ = 0.31; versus Geographic Stratum: R^2^ = 1.38; versus Sex: R^2^ = 4.87; versus Age Group: R^2^ = 5.68; versus Nutritional Status: R^2^ = 7.01; versus Practice of Physical Activity: R^2^ = 7.13; versus Sexual Maturity: R^2^ = 7.16.

****Exclusive Breastfeeding: R^2^ = 0.11; versus Regional Distribution: R^2^ = 0.39; versus Geographic Stratum: R^2^ = 1.48; versus Sex: R^2^ = 4.95; versus Age Group: R^2^ = 5.76; versus Nutritional Status: R^2^ = 7.10; versus Practice of Physical Activity: R^2^ = 7.22; versus Sexual Maturity: R^2^ = 7.25.

CI: confidence interval; Birthweight: low (<2500g), insufficient (2500 to 2999g), adequate (3000g to 3999g), and high (≥ 4.000g)^15^. Duration of pregnancy: <8 months and 9 to 10 months. Duration of exclusive breastfeeding: < 3 months, 3 to 6 months, and > 6 months^5^.

**Table 3:** Multiple linear regression between biological and behavioral factors in early life and percentage of lipids, carbohydrate and protein intake in Brazilian adolescents, ERICA 2013–2014.

	Percentage of Lipid Intake (%)					Percentage of Carbohydrate Intake (%)					Percentage of Protein Intake (%)				
Variables	Crude Analysis β (CI95%)	p	Adjusted Analysis* β (CI95%)	p	R^2^ (%)	Crude Analysis β (CI95%)	p	Adjusted Analysis** β (CI95%)	p	R^2^ (%)	Crude Analysis β (CI95%)	p	Adjusted Analysis*** β (CI95%)	p	R^2^ (%)
**Birthweight (g)**	** **				0.92	** **				0.94	** **				2.11
Adequate	1					1					1				
Low	-0.74 (-1.42;-0.06)	0.032	-0.59 (-1.23;0.05)	0.072		1.21 (.013;2.28)	0.027	1.25 (0.15;2.34)	0.025		-0.45 (-1.12;0.21)	0.180	-0.62 (-1.33;0.07)	0.079	
Insufficient	0.71 (-0.34;1.77)	0.186	0.69 (-0.39;1.78)	0.208		-0.96 (-2.27;0.34)	0.150	-1.03 (-2.34;0.27)	0.120		0.12 (-0.36;0.60)	0.622	0.19 (-0.28;0.68)	0.428	
High	-0.23 (-0.99;0.51)	0.540	-0.10 (-0.85;0.65)	0.790		0.13 (-0.79;1.06)	0.773	0.08 (-0.88;1.05)	0.864		0.28 (-0.42;0.48)	0.901	-0.02 (-0.52;0.47)	0.911	
No information	-0.52 (-1.06;0.00)	0.054	0.19 (-1.20;1.59)	0.786		0.55 (-0.10;1.22)	0.098	0.43 (-0.25;1.12)	0.216		-0.08 (-0.42;0.25)	0.625	-0.08 (-0.67;0.50)	0.776	
**Duration of Pregnancy (months)**	** **				0.79	** **				0.72	** **				2.05
≤ 8	1					1					1				
9 to 10	-0.37 (-1.73;0.98)	0.588	-0.32 (-1.69;1.05)	0.644		-0.06 (-1.69;1.57)	0.942	-0.11 (-1.74;1.51)	0.890		0.31 (-0.42;1.06)	0.402	0.32 (-0.38;1.03)	0.364	
No information	-0.86 (-2.06;0.32)	0.155	-0.00 (-1.08;1.06)	0.986		0.60 (-1.03;2.24)	0.471	0.44 (-1.15;2.05)	0.585		0.14 (-0.54;0.83)	0.678	-0.01 (-0.84;0.81)	0.964	
**Exclusive Breastfeeding (months)**	** **				0.98	** **				0.90	** **				2.06
< 3	1					1					1				
3 to 6	-0.01 (-0.53;0.50)	0.949	-0.02 (-0.54;0.48)	0.913		0.41 (-0.32;1.16)	0.272	0.45 (-0.29;1.20)	0.232		-0.35 (-0.80;0.08)	0.117	-0.38 (-0.85;0.08)	0.110	
> 6	1.29 (0.32;2.25)	0.009	1.32 (0.37;2.26)	0.006		-1.22 (-2.50;0.05)	0.062	-1.14 (-2.40;0.11)	0.074		-0.10 (-0.72;0.51)	0.748	-0.15 (-0.72;0.41)	0.599	
No information	-0.63 (-1.21;-0.05)	0.032	-0.34 (-1.01;0.31)	0.303		0.97 (0.13;1.81)	0.023	0.91 (0.07;1.76)	0.033		-0.32 (-0.75;0.11)	0.148	-0.36 (-0.86;0.13)	0.152	

* Results from generalized linear models between biological and behavior factors in early life and percentage of lipid intake adjusted by variables: Regional Distribution, Geographic Stratum, Sex, Mother’s Schooling, Socioeconomic Class, Practice of Physical Activity, and Sexual Maturity.

** Results from generalized linear models between biological and behavior factors in early life and percentage of carbohydrate intake adjusted by variables: Regional Distribution, Geographic Stratum, Sex, Socioeconomic Class, Nutritional Status, and Sexual Maturity.

*** Results from generalized linear models between biological and behavior factors in early life and percentage of protein intake adjusted by variables: Regional Distribution, Geographic Stratum, Sex, Mother’s Schooling, Nutritional Status, Practice of Physical Activity, and Sexual Maturity.

**β**: CI: confidence interval; Birthweight: low (<2500g), insufficient (2500 to 2999g), adequate (3000g to 3999g), and high (≥ 4.000g)^15^. Duration of pregnancy: <8 months and 9 to 10 months. Duration of exclusive breastfeeding: < 3 months, 3 to 6 months, and > 6 months^5^.

Adolescents who received exclusive breast breastfeeding for three to six months ingested 1.32% more lipids than those who received exclusive breast breastfeeding for less than three months (95% CI: 0.37 to 2.26, p = 0.006) (Table 3). This model explained 0.98% of the variation in the percentage of lipid intake. Adolescents born with low birth weight had 1.25% higher in carbohydrate intake (95% CI: 0.15 to 2.34, p = 0.025) compared to those born with adequate weight (Table 3). This model explained 0.94% of the variation in the percentage of carbohydrate intake in adolescence. No associations were found between the other independent variables and the proportion of lipid or carbohydrate intake (Table 3). Moreover, the percentage of protein intake was not associated with any early-life characteristics (Table 3).

## Discussion

The main findings of the present study were that low birth weight was associated with lower calorie intake and a greater consumption of carbohydrates in adolescents in comparison to those born with adequate weight. Moreover, infants who received exclusive breastfeeding for more than six months had a greater consumption of lipids in adolescence than those who were breastfed for less than three months.

The mean calorie intake and percentages of macronutrient intake in the present study were similar to values reported in other studies involving adolescents [23]. Regarding factors related to early life, low birth weight was significantly associated with lower energy intake and a greater percentage of carbohydrates in the diet of the adolescents. Moreover, exclusive breastfeeding for more than six months was associated with greater lipid consumption in adolescence. These findings diverge from data reported in cohort studies, in which individuals with low birth weight had greater calorie intake in adolescence and adulthood [3, 8, 34].

In a study conducted with identical and fraternal twins, Doornweerd & colleagues [8] found that those born with low birth weight consumed 115 kcal more and 0.7% more saturated fat than those with a higher birth weight even after adjusting for sex, age, and current weight. However, the authors did not find associations with the consumption of proteins and carbohydrates. Moreover, as no significant difference in total energy intake was found in the pairs of identical and fraternal twins, the authors speculated that such associations were independent of genetic factors. A possible explanation for such a finding may be related to the fact that the parents of children born with a low birth weight increase the offer of foods with a high calorie density in an effort to promote healthier growth and development in subsequent phases of life [9].

Another cross-sectional study showed that low birth weight was associated with increased fat intake in boys aged six to 12 years, but was not associated with total energy, protein, or carbohydrate intake in either sex [11]. The authors suggest that these gender-specific divergences in the results may be due to the involvement of two main physiological pathways related to short-term and long-term control of food intake (homeostatic and hedonic) that influence food preferences [11].

Despite the evidence cited above, other studies have found no significant associations between low birth weight and energy intake [6, 7]. Furthermore, scientific evidence on these associations is insufficient and the mechanisms are not completely clear [4]. Such results suggest that adolescents who were born with low birth weight likely presented more accelerated “catch up” weight in the first two years of life [35] and may therefore be more susceptible to subsequent metabolic complications, such as excess weight and body fat at the end of childhood and adolescence [35].

Regarding the association between low birth weight and a greater percentage of carbohydrates in the diet of the adolescents in the present study, studies conducted with children, adolescents, and adults with intrauterine growth restriction or exposed to hunger during gestation found that such individuals consume more palatable foods rich in carbohydrates and/or lipids than their peers [4, 6, 9, 34]. Although the present study only evaluated low birth weight and the duration of pregnancy, with no investigation of intrauterine growth restriction and “small for gestational age”, low birth weight can be considered a proxy measure to enable the comparison of these findings. Indeed, intrauterine growth restriction can cause low birth weight [4, 34] and therefore negatively influence food preferences and/or nutrient intake throughout life, which could partly explain the increased the risk of chronic noncommunicable diseases, such as obesity and metabolic syndrome [18].

Another study found that female adolescents who had been “small for gestational age” consumed more added sugar and less dietary fiber, vegetables, and essential fatty acids in comparison to those who were normal for gestational age, even after adjusting for socioeconomic variables of the parents, mother’s diet, and smoking during pregnancy [36]. However, this association was not found in boys [36].

Although the studies mentioned above show differences in food preferences, especially for sweet flavors [34, 37] in different sexes and age groups, they are similar with regards to the increased intake of foods with high energy density, rich in sugars and saturated fats. A possible mechanism for such an association would be related to the sensation of pleasure/reward during eating through the signaling of the hedonic system [37].

Adolescents who had breastfed exclusively for more than six months had greater lipid intake than those who had breastfed for less than three months. These results diverge from those found by Spaniol and collaborators [5], who showed that the intake of breast milk in the first and second year of life was associated with a lower likelihood of consuming ultra-processed foods (cookies with filling and crackers) and sugary drinks (soft drinks and artificial juices).

This finding may be explained by the notion that a mother’s diet can exert an influence on the composition and taste of breastmilk [38] and consequently lead to early exposure to different flavors, affecting food preferences throughout the phases of life [38, 39]. Another supposition for the increase in the consumption of lipids in adolescence would be the notion that individuals who received exclusive breastfeeding in the first months of life have a greater preference for fatty foods in the first years of life [38, 39] and it could by hypothesized that this behavior can extend into adolescence. Although the present results point to an increase in lipid intake in adolescence, studies in the literature found that the offer of breastmilk [40] and a longer period of breastfeeding were positively associated with a healthier eating pattern (higher intakes of fruits and vegetables in childhood) [41].

No associations were found between the duration of pregnancy and the food consumption outcomes analyzed in the present study. Few studies have addressed this issue. However, children with intrauterine growth restriction and those classified as “small for gestational age” have more eating problems than their peers [42, 43], as reported in the study by Kaseva et al. [4], who found that preterm infants with very low birth weight (≤ 1,500 g) had a lower consumption of fruits, vegetables, and dairy products compared to full-term infants.

The present findings may also be explained by the surrounding environment in which the family lives regarding health-related behavior, lifestyle, the duration of breastfeeding, the introduction of complementary foods, parental relations, composition of the diet, and socioeconomic status [12, 19, 38, 39, 44]. The discussion of this issue is somewhat complex, considering the limited body of evidence on the general population, especially adolescents, which hinders the interpretation of the possible late effects of early-life characteristics on eating behavior throughout the phases of life. Moreover, a high non-response rate (40% to 50%) occurred in the present study regarding biological and behavioral factors in early life, which may be attributed to recall bias. In an attempt to attenuate this limitation, “no information” categories were created for all variables with missing data.

Regarding the weak points and divergencies of this investigation compared to studies available in the literature, several factors exert an effect on associations between early-life characteristics and food consumption, such as the instrument used to assess food consumption [9], the different types of food consumption outcomes studied (calorie and macronutrient intake measured in grams or percentage) [6, 8], and the different phases of life analyzed [10, 36]. Moreover, most investigations with this approach have been experimental studies involving rats submitted to different malnutrition models [3, 45], which makes it difficult to extrapolate the results to humans, or cohort studies [4, 7, 34], which, unlike cross-sectional studies, enable the establishment of cause-and-effect relationships.

Thus, the present study has limitations that should be considered: 1) Studies on food consumption in humans are quite complex; due to its multifactorial nature, consumption is affected by biological and environmental factors, which may have exerted an influence on the results of the analyses performed in this study; 2) the instrument used for the assessment of food consumption (24-hour recall) increases the risk of recall bias and information bias, which regards the underestimation or overestimation of the consumption of some food groups on the part of the respondent; 3) the cross-sectional design increases the risk of reverse causality and does not enable establishing cause-and-effect relationships, as the participants were only evaluated in adolescence and could therefore be on a weight loss diet, which would result in a lower calorie intake compared to adolescents born with adequate weight; and 4) the results may have been influenced by the non-control of important confounding variables in the analyses, such as those related to the pregnancy (pregestational weight, work, income, smoking, and alcohol use during the pregnancy). Moreover, gestational age was not collected on the questionnaire used in the ERIKA Study. Therefore, a proxy measure was used (duration of pregnancy [main exposure] in number of months up to the birth).

The following were the strong points of this study: 1) The ERICA Study was considered the first national school-based cross-sectional survey to use the 24-recall method for the analysis of energy and macronutrient intake [23, 44]; 2) The present study analyzed two biological factors (low birth weight and pre-term birth) and one behavioral factor (exclusive breastfeeding) rather than the association of only one early-life factor with food intake, as found in most studies [4, 8, 10, 19]; 3) The evaluation of this issue in adolescents, as most studies have focused on other population subgroups, such as children [6, 7, 10, 42, 43] and adults [3, 4, 9]; 4) The execution of a cross-sectional study encompassing all geographic regions of Brazil. While cohort studies are more indicated for the evaluation of this issue, the high financial costs related to the long follow-up time as well as methodological and logistical difficulties make such studies far more difficult to execute [4]. Nonetheless, longitudinal studies should be conducted to evaluate the possible effects of early-life factors on food consumption and possible interactions in adolescents as well as a continual assessment of the effectiveness of intervention programs on the public health level for the promotion of healthy eating habits mediated by biological and behavioral factors in early life.

This study is important because it considered possible associations between early-life factors and food consumption. The literature has demonstrated a possible association mechanism between low birth weight and an increased risk of developing noncommunicable diseases, such as obesity and cardiovascular disease, due to changes in the energy balance associated with health-related behaviors, including food consumption [4]. We hope that this study can contribute greater knowledge regarding the influence of early-life characteristics on food consumption in adolescents, which could assist in improving child and maternal health care programs by encouraging the incorporation of healthy eating habits to attenuate adverse events in early life.
